# Is social camouflaging associated with anxiety and depression in autistic adults?

**DOI:** 10.1186/s13229-021-00421-1

**Published:** 2021-02-16

**Authors:** Laura Hull, Lily Levy, Meng-Chuan Lai, K. V. Petrides, Simon Baron-Cohen, Carrie Allison, Paula Smith, Will Mandy

**Affiliations:** 1 Research Department of Clinical, Educational and Health Psychology, University College London, 1-19 Torrington Place, London, WC1E 7HB UK; 2 Centre for Addiction and Mental Health and The Hospital for Sick Children, Department of Psychiatry, University of Toronto, Toronto, Canada; 3 Autism Research Centre, Department of Psychiatry, University of Cambridge, Cambridge, UK; 4 Department of Psychiatry, National Taiwan University Hospital and College of Medicine, Taipei, Taiwan; 5 London Psychometrics Laboratory, University College London, London, UK; 6 Present Address: Homerton University Hospital NHS Foundation Trust, London, UK

**Keywords:** Mental health, Camouflaging, Gender, Adults

## Abstract

**Background:**

There is inconsistent evidence for a clear pattern of association between ‘camouflaging’ (strategies used to mask and/or compensate for autism characteristics during social interactions) and mental health.

**Methods:**

This study explored the relationship between self-reported camouflaging and generalised anxiety, depression, and social anxiety in a large sample of autistic adults and, for the first time, explored the moderating effect of gender, in an online survey.

**Results:**

Overall, camouflaging was associated with greater symptoms of generalised anxiety, depression, and social anxiety, although only to a small extent beyond the contribution of autistic traits and age. Camouflaging more strongly predicted generalised and social anxiety than depression. No interaction between camouflaging and gender was found.

**Limitations:**

These results cannot be generalised to autistic people with intellectual disability, or autistic children and young people. The sample did not include sufficient numbers of non-binary people to run separate analyses; therefore, it is possible that camouflaging impacts mental health differently in this population.

**Conclusions:**

The findings suggest that camouflaging is a risk factor for mental health problems in autistic adults without intellectual disability, regardless of gender. We also identified levels of camouflaging at which risk of mental health problems is highest, suggesting clinicians should be particularly aware of mental health problems in those who score at or above these levels.

## Background

Autism spectrum disorder (hereafter ‘autism’) is a neurodevelopmental condition that affects how a person relates to others and experiences the world. To meet diagnostic criteria for autism, an individual must display difficulties with social interaction and communication, in addition to restricted interests and repetitive behaviours [1]. Across the lifespan, autistic people are at very high risk of mental health problems. In a community sample of young autistic people aged between 10 and 14 years, 71% were found to meet criteria for at least one mental health disorder, with 41% having additional comorbidities [2]. Similarly high rates of mental health problems are consistently found in autistic adults [3–6]. Further, compared to the general population, autistic adults experience higher rates of suicidal ideation and are more likely to die by suicide [7–10].

While high rates of mental health problems in autism persist across childhood, adolescence, and adulthood, the specific nature of these difficulties changes over time [11]. Among children, anxiety disorders, attention deficit hyperactivity disorder (ADHD), and oppositional defiant disorder (ODD) predominate [2, 12]. By adulthood, this profile of risk has shifted. The prevalence of anxiety problems remains high with the transition to adulthood, but rates of externalising problems (ADHD, ODD) reduce, and risk of depression greatly increases [3, 4, 11]. As a result, depression and anxiety are the two most common psychiatric problems for autistic adults [8, 13–15].

Mental health difficulties have a substantial impact upon the well-being and life opportunities of autistic people. Their mental health problems are associated with lower social and adaptive functioning [5], educational and employment difficulties [16, 17], higher parental stress [18], poor quality of life [19, 20], and suicidality [21]. Despite this, the evidence base on how to treat mental health problems in autism is meagre, and most autistic people with mental health problems report receiving inadequate help for these difficulties [22]. Reflecting this, research into mental health problems has been identified as a key priority for research across a variety of stakeholder groups [23, 24].

Efforts to improve mental health services for autistic people are constrained by a current lack of understanding about what causes and maintains their mental health problems. Crucially, there is emerging evidence that some risk processes may be specific to autistic populations, such that it may not be effective simply to transpose findings from non-autistic populations to inform the treatment for autistic people. For example, in the general population, anorexia nervosa, by definition, is driven by weight and body shape concerns [25]. But many autistic women diagnosed with this eating disorder do not show a desire for thinness and body image preoccupations: their eating psychopathology is often driven by autism-specific factors, such as sensory aversion to food texture and controlled eating and exercise as a way to minimise uncertainty [26–30]. Similarly, specific cognitive biases that underpin anxiety problems in non-autistic people may be less prominent in highly anxious autistic people [31]. Instead, autism-specific risk factors, such as an intolerance of uncertainty and sensory overload, are important in the development and maintenance of clinically severe anxiety in autism [32].

In the current study, we seek to investigate a putative autism-specific risk factor for mental health problems of autistic people, namely social camouflaging. Social camouflaging is defined as ‘the use of strategies by autistic people to minimise the visibility of their autism in social situations’ [33, p. 819]. These strategies can involve masking autistic characteristics, for example by deliberately suppressing stimming behaviour in public [34]. Social camouflaging also encompasses compensatory behaviour, whereby autistic people develop behavioural repertoires to help them manage social situations. Common examples of compensatory behaviours include learning how to use specific non-verbal social behaviours, such as eye contact, and developing scripts that can be employed to navigate interactions [34, 35]. Social camouflaging is a widespread element of the lived experiences of autistic people, especially among those who are female [36] and those who have intelligence in the average range or above [37].

There is emerging evidence that social camouflaging may be a risk factor for anxiety and depression in autism. Studies based on qualitative analysis of in-depth interviews have shown that autistic people often feel their camouflaging renders them more vulnerable to mental health problems [38–40]. The relevant qualitative literature suggests a number of potential mechanisms whereby camouflaging could lead to poor mental health. Camouflaging is very commonly reported to be exhausting [34, 40] and stressful [39]. Furthermore, the habitual practice of pretending not to be autistic can erode a person’s sense of identity [34] and can lead to their needs being misunderstood or overlooked entirely [38]. In addition, Cage and colleagues have suggested that camouflaging may mediate the relationship between social stressors (e.g. bullying, lack of autism acceptance) and subsequent anxiety and depression [39].

While qualitative literature suggests the possibility that camouflaging is a risk factor for internalising problems, attempts to test this idea using quantitative methods have so far yielded somewhat inconsistent results. There have been reported associations between social camouflaging and anxiety difficulties in adults [41] and in children [37]; as yet there has been no investigation of camouflaging and mental health in adolescence, although this is an especially critical time period for the development of mental health problems. Two other studies looked for and did not find an association between social camouflaging and anxiety [36, 39]. These two investigations were powered to detect moderate and above effects (rs ≥ 0.34), so one possibility is that they missed a true, smaller relationship. Another possibility is that a more fine-grained investigation of anxiety difficulties is needed [37]; many studies have reported on anxiety broadly, without contrasting social anxiety and generalised anxiety symptoms. As social anxiety and generalised anxiety are both common in the autistic population [11] and may have different causes and responses to intervention [32], it is important to consider whether they also have different associations with camouflaging. For example, it may be that social camouflaging predicts social anxiety, but not generalised anxiety.

Quantitative findings on social camouflaging and depression have also been inconsistent. In one study, Cage and colleagues [39] found that participants who spontaneously reported camouflaging were most likely to experience depression. However, in a subsequent, larger study using a more extensive and robust measure of camouflaging, they found no relationship between camouflaging and depression [41]. Lai et al. [36] found that camouflaging predicted depressive symptoms in men, but not women. In a sample of women with high levels of autistic traits, camouflaging was found to predict psychological distress (a combined measure of anxiety, depression, and stress) and was significantly correlated with suicidal behaviours in a subsample who reported higher than average camouflaging [42]. This suggests the need for future investigations to consider moderating effects of sex/gender. We note that for this paper we consider the impacts of sex and gender as potentially distinct, but also interactive, and so refer to sex/gender generally unless talking about the specific impact of either biological sex or gender identity. Previous research has also demonstrated that camouflaging is positively correlated with autistic traits [33]; however, not all previous studies into camouflaging and mental health have controlled for autistic traits in analyses. As autistic traits are commonly related to mental health problems [6] and to levels of camouflaging [33], it is essential for studies to control for any potential covariate effect.

In addition to seeking to enhance understanding of whether there is an association between camouflaging and mental health, we aim to make a practical estimate of the risk of mental health problems associated with camouflaging that could be used to inform clinical practice. There are undoubtedly some benefits of camouflaging for some autistic people [34, 39], but the associations described previously suggest that, at a certain level, the costs may outweigh the benefits. How much camouflaging is ‘too much’, when it comes to mental health risks, for autistic adults? Is there a level of camouflaging which may be particularly harmful for some people? This information may be helpful for identification of those who might be at heightened risk for mental health problems as a result of camouflaging.

In summary, social camouflaging is a plausible candidate risk factor contributing to the high rates of anxiety and depression experienced by autistic people. Nevertheless, studies looking at associations between camouflaging and internalising problems have so far yielded inconsistent results, and this may be due to methodological factors. In the current study, we aim to address some of these methodological factors by: (a) recruiting a sample of sufficient size to detect small effects and test for moderating effects of sex/gender; (b) distinguishing between social and generalised anxiety; and (c) controlling for severity of autistic traits. We will address the following research questions:

1. Among autistic adults, is higher self-reported camouflaging associated with higher rates of social anxiety, generalised anxiety and depression symptoms, controlling for autistic trait intensity?
2. Does gender moderate the relationship between camouflaging and internalising mental health difficulties (social anxiety, generalised anxiety, and depression)?
3. Is there a ‘sub-optimal’ level of camouflaging with a greatest risk of mental health problems? What is the risk of mental health problems at different levels of camouflaging?

## Methods

### Participants

Participants (*N* = 305, ages ranging from 18 to 75 years) were from a project investigating social camouflaging in autistic adults [33]. They were recruited from the Cambridge Autism Research Database (an established database of formally diagnosed autistic adults in the UK; https://www.autismresearchcentre.net/), and via social media and adverts disseminated by relevant UK-based autism charities. Participants responded to an advert asking for autistic people to complete an online study of ‘social behaviours and well-being’, for which no financial reimbursement was offered. To be included in the current study, participants had to report an official autism diagnosis and the type of healthcare professional who gave this diagnosis. In this context, autism diagnoses included ‘autism spectrum disorder’, ‘autism’, ‘Asperger’s syndrome/disorder’, ‘atypical autism’, ‘autism spectrum condition’, or ‘pervasive developmental disorder, not otherwise specified’. People who reported being self-diagnosed, diagnosed with ‘autistic traits’ or who reported a diagnosis from someone other than a healthcare professional were excluded from the current study. We aimed to test whether gender moderates the relationship between camouflaging and internalising problems. We asked participants to report their gender identity and whether this differed from their gender assigned at birth; 22 participants self-identified as a different gender (including a non-binary gender) to their gender assigned at birth. Gender difference analyses in this study are conducted based on self-identified gender.

As a sensitivity analysis, we also grouped participants based on whether their gender identity corresponded with their gender assigned at birth (i.e. cisgender female, cisgender male, and transgender/non-binary individuals) and repeated the gender difference analyses in these groups.

Participants were asked to report whether they had ever received a formal psychiatric diagnosis of an anxiety disorder, depression, social anxiety/social phobia, or other conditions, in addition to their nationality, native language, and occupation. In the total sample, 55.4% were British, 21.6% were North American, 11.5% were European, 4.3% were Australasian, and 7.8% were from Asia, the Middle East, or South America. The proportion of the total sample who reported that their first language was English was 85.9%. With regard to occupation, 52.4% of the total sample were in full- or part-time paid employment, 15.7% were studying, 11.8% were unemployed, 10.8% were homemakers, carers, or volunteers, while 9.3% were disabled, retired, or did not respond.

Table 1 shows the characteristics of the study participants, based on the total sample and broken down by gender. Post hoc sensitivity power analyses demonstrated that in the total sample (*N* = 305), we had adequate (80%) power to detect small (*r* ≥ 0.12) effect sizes, with two-tailed alpha set at 0.05. Among the males (including cisgender and transgender males; *N* = 104), we had adequate power to detect small-to-medium effects (*r* ≥ 0.28), and among the females (including cisgender and transgender females; *N* = 181), we had power to detect small effects (*r* ≥ 0.21). In non-binary participants (*N* = 18), we had power to detect only large effects (*r* > 0.60).

**Table 1:** Participant characteristics and mean scores (with 95% confidence intervals) on all variables

	Total sample (*N* = 305)	Female subsample (*N* = 181)	Male subsample (*N* = 104)	Non-binary gender subsample (*N* = 18)
Age (years)	41.90 (40.37, 43.43)	39.76 (37.93, 41.61)	47.46 (44.77, 50.15)	36.00 (30.69, 41.31)
Generalised anxiety diagnosis (%)	173 (56.7%)	107 (59.1%)	52 (50%)	13 (76.5%)
Depression diagnosis (%)	166 (54.4%)	103 (56.9%)	49 (47.1%)	13 (76.5%)
Social Anxiety Disorder/Social Phobia diagnosis (%)	7 (2.3%)	6 (3.3%)	1 (0.9%)	0
Other psychiatric condition				
1 additional diagnosis	77 (25%)	50 (28%)	22 (21%)	5 (28%)
2 additional diagnoses	44 (14%)	28 (15%)	11 11%)	5 (28%)
3 or more additional diagnoses	29 (9%)	18 (10%)	8 (8%)	3 (11%)
Autistic traits (BAPQ)	4.32 (4.25, 4.39)	4.37 (4.28, 4.46)	4.23 (4.08, 4.38)	4.35 (4.15, 4.55)
Camouflaging (CAT-Q)	111.11 (109.10, 113.12)	113.96 (111.49, 116.43)	106.38 (102.73, 110.03)	110.29 (103.11, 117.47)
Social anxiety (LSAS)	82.53 (79.20, 85.87)	87.09 (82.84, 91.24)	75.15 (69.04, 81.26)	80.06 (70.79, 89.33)
Generalised anxiety (GAD)	9.79 (9.10, 10.49)	10.09 (9.23, 10.96)	9.15 (7.90, 10.40)	10.44 (7.36, 13.52)
Depression (PHQ)	11.74 (10.97, 12.51)	11.85 (10.91, 12.79)	11.08 (9.67, 12.49)	14.5 (11.17, 17.83)

*CAT-Q* Camouflaging Autistic Traits Questionnaire, *BAPQ* Broad Autism Phenotype Questionnaire, *LSAS* Liebowitz Social Anxiety Scale, *PHQ* Patient Health Questionnaire, *GAD* Generalised Anxiety Disorder Assessment. (NB. Two participants chose not to report their gender and so are included in the total sample only.)

### Measures

#### Camouflaging of Autistic Traits Questionnaire (CAT-Q)

Social camouflaging was measured by self-report, using the 25-item Camouflaging of Autistic Traits Questionnaire (CAT-Q; 33). This was developed based on a qualitative analysis of autistic people’s experiences [34], covering the following three elements of camouflaging: masking behaviours (e.g. ‘I monitor my body language or facial expressions so that I appear interested by the person I am interacting with’); compensation (e.g. ‘I have spent time learning social skills from television shows and films, and try to use these in my interactions’); and assimilation (‘I have to force myself to interact with people when I am in social situations’). Responses are given on a seven-point Likert scale, ranging from ‘strongly disagree’ to ‘strongly agree’. All items are summed to yield a total camouflaging score, with higher scores indicating higher levels of self-reported camouflaging. Possible scores range from 25 (no endorsement of camouflaging strategies) to 175 (strong endorsement of camouflaging strategies). The total camouflaging score of the CAT-Q has strong reliability: internal consistency (Cronbach’s alpha = 0.93) and test–retest reliability (ICC = 0.77) are high [33]. In the present study, internal consistency was acceptable (*α* = 0.79) in the total sample.

#### Broader Autism Phenotype Questionnaire (BAPQ)

Autism symptom intensity was assessed by the self-report version of the BAPQ, which has 36 items covering social reciprocity, social communication, and rigidity. This measure was originally designed to measure the intensity of autistic traits associated with a genetic liability to autism [43], but has come to be more widely used as a general-purpose dimensional measure of autistic characteristics in the general population and in autistic people [44, 45]. The BAPQ has strong reliability, good sensitivity, and specificity and measures the full range of autistic symptoms, without ceiling effects in autistic populations [33, 43, 46]. In the total sample, internal consistency was high (*α* = 0.92).

#### Social Anxiety Scale (LSAS)

This 24-item self-report questionnaire was designed to measure social anxiety symptoms in the general population [47]. It asks participants to imagine various social situations (e.g. ‘meeting strangers’) and to report how much fear this would engender, and how often they would avoid the situation. The LSAS has good test–retest reliability and discriminant validity [48]. A score of 30 or greater was identified as a clinical cut-off for social anxiety disorder [49]. In the current sample, internal consistency was high (*α* = 0.96) for the total scale (combined fear and avoidance questions).

#### Generalised Anxiety Disorder (GAD-7)

We used this seven-item self-report questionnaire to quantify generalised anxiety symptoms experienced in the last 2 weeks [50]. The GAD-7 demonstrates good sensitivity and specificity (Spitzer et al. 2006) and had high internal consistency in the current sample (*α* = 0.92). Scores of 10 or above are associated with moderate generalised anxiety, and 10 has been identified as a clinical cut-off [50].

#### Patient Health Questionnaire (PHQ-9)

The PHQ-9 is a widely used self-report measure of depressive symptoms in the last 2 weeks [51]. It has good sensitivity and specificity for major depressive disorder [51] and has been validated with autistic adults [52]. Internal consistency in the current study was good (*α* = 0.89). A score of 10 or greater has been identified as a clinical cut-off for major depressive disorder [51].

### Procedure

Ethical approval for the study was obtained from University College London Research Ethics Committee, reference 7475/002, and the study was carried out in accordance with the Declaration of Helsinki. Participants followed a link to the questionnaires, which were administered as an online survey using Qualtrics software. Initially, they were presented with an information sheet and the option to complete a consent form. Only those who completed the consent form could access the survey, which comprised demographic questions, the CAT-Q, the BAP-Q, the LSAS, the GAD-7, and PHQ-9, as part of a broader battery of online questionnaires.

### Analyses

As some participants were missing responses on some measures, multiple imputation was performed to reduce potential for bias by maximising the usable proportion of the sample. Nine participants were missing some scores on the BAPQ (3% of total sample), 21 participants (7%) were missing some scores on the LSAS, and 18 participants (6%) for each of the GAD and the PHQ. Multiple imputation was performed using the Hmisc function in R, with estimates from five imputations pooled to produce the imputed data, which were integrated with the original data to produce a final dataset of 305 participants with complete responses.

Means were calculated for all variables included in the regressions for the total sample and female, male, and non-binary subsamples (Table 1). Bivariate correlations between all variables were calculated for the total sample (Additional file 1: Table 1), male and female subsamples (Additional file 1: Table 2), and non-binary subsample (Additional file 1: Table 3).

**Table 2:** Hierarchical regression models predicting generalised anxiety (Model 1), depression (Model 2), and social anxiety (Model 3) from age, autistic traits, and camouflaging in the total sample (*N* = 305)

	Variable	*B*	*β*	*p*	DF	*F*	*p*	*R*^2^Adj	Delta *R*^2^
Model 1 (generalised anxiety)									
Step 1					2, 302	31.99	< .001	0.17	
	Age	− 0.07	− 0.15	.013					
	BAPQ	3.71	0.40	.006					
Step 2					3, 301	28.16	< .001	0.21	0.04
	Age	− 0.05	− 0.11	.042					
	BAPQ	3.24	0.35	< .001					
	CAT-Q	0.08	0.22	< .001					
Step 3					4, 300	21.08	< .001	0.21	0.00
	Age	− 0.05	− 0.11	0.04					
	BAPQ	3.22	0.35	< .001					
	CAT-Q	0.11	0.33	0.42					
	CAT-Q^2^	− 0.01	− 0.11	0.79					
Model 2 (Depression)									
Step 1					2, 302	27.41	< .001	0.15	
	Age	− 0.09	− 0.18	< .001					
	BAPQ	3.68	0.36	< .001					
Step 2					3, 301	20.18	< .001	0.16	0.01
	Age	− 0.08	− 0.16	.004					
	BAPQ	3.39	0.33	< .001					
	CAT-Q	0.05	0.12	.026					
Step 3					4, 300	15.09	< .001	0.16	0.00
	Age	− 0.08	− 0.16	.004					
	BAPQ	3.39	0.33	< .001					
	CAT-Q	0.06	0.17	0.70					
	CAT-Q^2^	− 0.01	− 0.04	0.92					
Model 3 (Social Anxiety)									
Step 1					2, 302	89.67	< .001	0.37	
	Age	− 0.29	− 0.14	.004					
	BAPQ	26.90	0.60	< .001					
Step 2					3, 301	70.36	< .001	0.41	0.04
	Age	− 0.21	− 0.10	.034					
	BAPQ	24.76	0.55	< .001					
	CAT-Q	0.34	0.21	< .001					
Step 3					4, 300	52.65	< .001	0.41	0.00
	Age	− 0.21	− 0.10	.036					
	BAPQ	24.67	0.55	< .001					
	CAT-Q	0.55	0.33	.358					
	CAT-Q^2^	− 0.01	− 0.12	.829					

The quadratic term of camouflaging is added at Step 3

*BAPQ* Broad Autism Phenotype Questionnaire, *CAT-Q* Camouflaging Autistic Traits Questionnaire, *β* standardised beta

**Table 3:** Hierarchical regression models predicting generalised anxiety (Model 1a), depression (Model 2a), and social anxiety (Model 3a) from age, gender, autistic traits, and camouflaging in the total male and female sample (N = 286)

	Variable	*B*	*β*	*p*	DF	*F*	*p*	*R*^2^Adj	Delta *R*^2^
Model 1a									
Step 1					4, 282	21.31	< .001	0.22	
	Age	− 0.05	− 0.11	.046					
	Gender	− 0.46	− 0.04	0.516					
	BAPQ	3.40	0.37	< .001					
	CAT-Q	0.07	0.22	< .001					
Step 2					5, 281	17.88	< .001	0.22	0.00
	Age	− 0.05	− 0.11	.040					
	Gender	7.02	0.55	.087					
	BAPQ	3.33	0.37	< .001					
	CAT-Q	0.18	0.53	.003					
	CAT-Q * Gender	− 0.07	− 0.15	.065					
Model 2a									
Step 1					4, 282	14.06	< .001	0.15	
	Age	− 0.07	− 0.15	.011					
	Gender	− 0.58	− 0.04	0.472					
	BAPQ	3.44	0.34	< .001					
	CAT-Q	0.05	0.12	.038					
Step 2					5, 281	11.55	< .001	0.16	0.01
	Age	− 0.07	− 0.15	.009					
	Gender	4.98	0.35	.293					
	BAPQ	3.39	0.34	< .001					
	CAT-Q	0.13	0.33	.077					
	CAT-Q * Gender	− 0.05	− 0.10	.234					
Model 3a									
Step 1					4, 282	54.16	< .001	0.43	
	Age	− 0.20	− 0.09	.054					
	Gender	4.56	0.07	.123					
	BAPQ	25.27	0.57	< .001					
	CAT-Q	0.32	0.19	< .001					
Step 2					5, 281	43.20	< .001	0.43	0.00
	Age	− 0.20	− 0.09	.055					
	Gender	0.15	0.01	.993					
	BAPQ	25.31	0.57	< .001					
	CAT-Q	0.25	0.15	.333					
	CAT-Q * Gender	0.04	0.02	.796					

*BAPQ* Broad Autism Phenotype Questionnaire, *CAT-Q* Camouflaging Autistic Traits Questionnaire, *β* standardised beta

### Research Question 1

Hierarchical linear multiple regressions were run to test whether camouflaging was a significant predictor of mental health problems (generalised anxiety, depression, and social anxiety), while controlling for age and level of autistic traits. Age and autistic traits were entered at Step 1, with camouflaging added at Step 2.

Three regression models were run in the total sample, testing the association between camouflaging and generalised anxiety (Model 1), depression (Model 2), and social anxiety (Model 3).

### Research Question 2

To examine the impact of gender on the relationship between camouflaging and mental health, hierarchical linear regressions were run with binary gender (male and female) as a baseline predictor (alongside age, autistic traits, and camouflaging) at the first stage. An interaction term was then added to the models (Camouflaging*Gender) as a second stage.

There were no significant correlations between camouflaging and the mental health problems in the non-binary subsample, likely due to the small number of participants. As such, no regressions were run in this subsample and correlational results only are discussed.

### Research Question 3

To examine whether there is a ‘suboptimal range’ of camouflaging—i.e. a specific range in which camouflaging is most strongly associated with poor mental health, a quadratic term for camouflaging (CAT-Q^2^) was added at step 3 to the regressions described for Research Question 1.

Probabilities of scoring above the clinical cut-off point for each mental health problem were calculated for multiple scores on the CAT-Q, controlling for age and autistic traits, in the total sample.

All analyses were run in R (version 3.6.3) and RStudio (version 1.3.159).

## Results

### Research Question 1: Does camouflaging predict mental health difficulties, while controlling for age and autistic traits?

Results of three linear regressions predicting generalised anxiety, depression, and social anxiety from age, autistic traits, and camouflaging are reported in Table 2 (Models 1, 2, and 3).

In all three models, camouflaging significantly predicted the mental health outcome, beyond the significant contributions of age and autistic traits. Greater camouflaging scores predicted greater generalised anxiety, depression, and social anxiety. The unique contribution of camouflaging (Step 2 in each model) was relatively small, but was greatest in Models 1 (generalised anxiety) and 3 (social anxiety).

### Research Question 2: Does gender moderate the relationship between mental health and camouflaging?

To address this question, hierarchical linear regressions were run, with binary gender (female and male) added as an additional predictor at step one and an interaction between gender and camouflaging added as an additional step. The results are presented in Table 3.

No main effect of gender was found in any model. The interaction between gender and camouflaging was not significant in any model. Because of the lack of significance observed here, no further analyses were conducted to examine gender differences. When analyses were repeated with participants grouped by gender identity congruence (cisgender female, cisgender male, and transgender/non-binary individuals), no difference in results was found; see Additional file 1: Table 4 for a summary of these findings.

**Table 4:** Probability of scoring above the clinical cut-off point for generalised anxiety (GAD-7), depression (PHQ-9), and social anxiety (LSAS), at a range of total camouflaging scores

	Probability of scoring above cut-off		
	GAD > = 10	PHQ > = 10	LSAS > = 30
CAT-Q = 25	.04	.13	.16
CAT-Q = 50	.09	.22	.44
CAT-Q = 75	.19	.36	**.76**
CAT-Q = 100	.37	.51	.92
CAT-Q = 125	**.60**	**.67**	.98
CAT-Q = 150	.79	.80	.99
CAT-Q = 175	.90	.88	.99

Probabilities with the greatest increase for each mental health problem are in bold

*GAD* Generalised Anxiety Disorder Assessment, *PHQ* Patient Health Questionnaire, *LSAS* Liebowitz Social Anxiety Scale, *CAT-Q* Camouflaging Autistic Traits Questionnaire

### Research Question 3: What is the risk of mental health problems at different levels of camouflaging?

Table 2 also demonstrates the results of nonlinear regressions, where camouflaging was included as a quadratic variable (Step 3 in Models 1, 2, and 3).

When a quadratic term was included in the model, it was not a significant predictor of any mental health condition, suggesting that there is a linear association between camouflaging score and symptoms of generalised anxiety, depression, and social anxiety.

The probability of scoring at or above the clinical cut-off point for generalised anxiety (GAD7), depression (PHQ9), or social anxiety (LSAS) was calculated for multiple scores on the CAT-Q (in units of 25 points from the minimum score, 25, to the maximum score, 175), while controlling for age and autistic traits. Results from the total sample are presented in Table 4.

## Discussion

This study aimed to examine the association between self-reported camouflaging and mental health problems (generalised anxiety, depression, and social anxiety) in autistic adults. Gender differences in these relationships were also investigated, and we examined risk of clinically meaningful mental health problems at different levels of self-reported camouflaging.

In the total sample of autistic adults, camouflaging significantly positively predicted scores on measures of generalised anxiety, depression, and social anxiety, even when age and level of autistic traits were included as control variables. The strongest relationships found were for generalised and social anxiety.

It is important to note that these analyses are cross-sectional, and therefore, we cannot determine which of the mental health conditions and camouflaging behaviours developed first and affected the others. For example, it may be that pre-existing mental health difficulties lead autistic adults to camouflage more, perhaps to hide their additional needs, or to increase their ability to access mental-health-specific support. Longitudinal research is essential to determine the nature of any causal relationships. However, as qualitative research based on the lived experience of autistic people suggests that camouflaging does contribute to the development of mental health problems [34], our discussion will focus on exploring this direction of association.

Our findings support previous studies examining the association between camouflaging and mental health problems and add to a growing body of evidence that camouflaging may be harmful to autistic adults (although some individuals also report some positive benefits of camouflaging; [34, 53]). There are several potential mechanisms through which this relationship may develop. First, camouflaging has consistently been reported to be exhausting [34, 40, 54]. It has also been proposed as a cause of burnout, which reduces autistic adults’ quality of life and their ability to live independently [53, 55]. The mental and physical efforts needed to camouflage may reduce individuals’ capacity to deal with negative emotions and exacerbate existing mental health problems. Second, camouflaging may reduce feelings of authenticity and acceptance [34, 38]. For instance, autistic adults who spontaneously reported camouflaging their autism were less likely to report acceptance from others, which was itself associated with depression and stress [39]. Similarly, autistic adults who camouflage more report feeling a reduced sense of belonging, and higher levels of suicidality [21]. Individuals who camouflage more may have lower self-esteem and fewer close relationships, both of which are associated with poor mental health outcomes [56, 57].

Negative outcomes of camouflaging have been previously reported by autistic adults of multiple genders [34, 53]. In the present study, gender (binary; male and female) was added to these models as a potential moderator. No main effects were found when camouflaging was included as a predictor, nor any statistically significant interactions between gender and camouflaging. This result was strengthened by our inclusion of sensitivity analyses, repeating the gender difference analyses but grouping participants based on their gender identity congruency (i.e. cisgender female, cisgender male, and transgender/non-binary), where no main or interactive effect of gender was found. Our findings suggest that the association between camouflaging and mental health (specifically, generalised anxiety, depression, and social anxiety) does not differ for autistic men and women.

These results contrast with the only known previous study comparing the association between camouflaging and mental health separately in autistic men and women [36], which found that camouflaging was significantly correlated with depression in autistic men, but not women. The inconsistency between these findings may be partly due to methods used; Lai and colleagues used a discrepancy method to quantify camouflaging, which may capture characteristics related to the external success of camouflaging strategies. In contrast, the present study used a self-report method where participants reported on their use of camouflaging strategies, regardless of success. It may be that the use of camouflaging strategies has a negative impact on mental health regardless of one’s gender, but that autistic men experience poorer outcomes of ‘successful’ camouflaging (i.e. camouflaging which impacts external behaviour) compared to women. An alternative explanation is that the study by Lai and colleagues may have been underpowered to detect an association between camouflaging and depression in autistic women, as only 30 autistic women were included in the study. The predictive effect of camouflaging on depression was small in the present study, with the addition of camouflaging increasing the variance explained by only a small amount more than the variance explained by age and autistic traits. It may be that camouflaging plays less of a role in the development of depression in autism, compared to generalised and social anxiety. Further replication using multiple measures of camouflaging, in sufficiently powered samples, would provide more evidence to establish a conclusive answer.

In addition, research examining gender in relation to camouflaging and mental health needs to specifically address the experiences of people with non-binary gender identities; the present study included a small number of non-binary people but not enough to compare statistically. Correlational analyses of camouflaging with mental health problems in the non-binary subsample were not significant (see Additional file 1: Table 3), although it is possible that significant associations could be identified in a larger, adequately powered sample.

Although there is some evidence suggesting the impact of camouflaging may be moderated by sex/gender [58], the reason for this is not well understood. Some researchers and autistic people have proposed that there are different social expectations and pressures across genders, such that autistic individuals who are perceived or socialised as female may experience greater pressure to fit in and appear neurotypical [38], thereby motivating camouflaging. However, the emerging evidence of the impact of camouflaging on mental health in adult males and the results of the present study suggest that social expectations driving camouflage also exist for those perceived or socialised as male, although these may differ in content. Sex-specific mechanisms have also been proposed which may influence the development of neural processes involved in camouflaging, such as activation of neural areas associated with self-representation in autistic females, but not males [59].

It could also be suggested that social expectations and pressures resulting in camouflaging may differ according to how others perceive and respond to an individual; therefore, transgender individuals may have more similar camouflaging experiences to non-binary adults, compared to cisgender men/women. To test this, we repeated the analyses of gender differences, grouping participants according to whether their gender identity aligned with the sex they were assigned at birth. No significant effect of sex/gender identity congruence was found in these analyses (see Additional file 1: Table 4), suggesting that camouflaging has a similarly negative impact across gender identities, regardless of whether an individual is cisgender or transgender. However, the total number of transgender and non-binary participants (*N* = 22) was still underpowered to detect small or moderate associations, and so these analyses should be repeated in a larger sample before firm conclusions can be drawn. Ultimately, it is likely that the most fruitful investigations will explore the interactions between sex, gender, and the environment(s) in which an individual develops [60], to better understand why some autistic people camouflage more than others, and why this may impact them in different ways.

The final aim of this study was to identify the risk of mental health problems at different levels of camouflaging, in order to provide some guidance for those seeking to support someone who camouflages. Camouflaging was not a significant predictor in a nonlinear model for any mental health condition, suggesting that the association between camouflaging and poor mental health in autism is linear. In other words, the more an autistic individual camouflages, the more likely they are to experience symptoms of generalised anxiety, depression, and social anxiety, and this relationship exists across the full spectrum of camouflaging level.

Despite this finding, it would be helpful to identify levels of camouflaging at which individuals might meet clinical cut-offs for mental health conditions, to prioritise screening for these conditions. The probability of scoring above the clinical cut-off on measures of generalised anxiety, depression, and social anxiety was calculated for the entire sample. It is not possible to identify overall cut-off scores for clinically meaningful levels of camouflaging on the CAT-Q, as the impact of camouflaging appears to vary extensively depending on the individual and the situation in which they camouflage [34, 41]. However, these analyses do allow us to identify scores at which individuals can be considered at greater risk of specific mental health conditions.

Individuals scoring 125 and above on the CAT-Q showed the greatest relative increase in risk of generalised anxiety and depression, while individuals scoring 75 or higher showed the greatest relative increase for social anxiety. It is important to note that all three measures used to assess generalised anxiety, depression, and social anxiety are screening tools and so scores above the threshold do not necessarily translate into a clinical diagnosis of any condition. These estimates should not be considered as either diagnostic or deterministic; instead, they reflect levels of camouflaging at which the risk of specific mental health conditions may increase the most. Individuals who score at or above these levels may be at particularly heightened risk of having clinically significant levels of mental health conditions, and so should be assessed clinically, if they have not already. However, it is important to point out the presence of camouflaging, regardless of the extent, has been associated with higher scores on measures of mental health conditions, and so, where possible, autistic individuals should be assessed for mental health difficulties regardless of their level of camouflaging.

These findings have important implications for considering the role of camouflaging strategies. Autistic individuals’ attitudes to camouflaging vary considerably; while many dislike camouflaging and either choose or want to reduce their levels of camouflaging, others find it helpful or would like to be able to camouflage more [34]. Our findings suggest that promoting more camouflaging generally may lead to increased risk of mental health conditions across sexes and genders. An alternative approach may be to identify components of camouflaging strategies and evaluate their impact for that particular person, to determine which strategies lead to distress or negative mental states, and which are considered beneficial by the person. If an autistic person would like support in developing their camouflaging strategies, it may be useful to introduce specific strategies and monitor their impact on mental health over time.

We still do not know whether there are specific camouflaging strategies or components which have a greater impact on mental health than others; future research should examine the association between individual subscales of the CAT-Q (Compensation, Masking, and Assimilation) and specific mental health conditions. Different strategies may have different levels of success, which may contribute to a positive impact on their mental health. One strategy (such as forcing oneself to make eye contact) may be relatively easy and successful for one individual and so allow them to get through social interactions smoothly; on the other hand, it may be painful and effortful for another person, distracting them from other social behaviours, so that it has an overall negative impact on their social abilities and causes them distress when used regularly. Some autistic adults may have been explicitly taught social skills, particularly those who were diagnosed in childhood; it is still not clear how much camouflaging overlaps with the use of learned social skills in autism. However, our results do suggest that autistic adults experiencing mental health difficulties should be encouraged to reflect on and discuss their use of camouflaging, which may play an important role in the development or persistence of their mental health problems.

## Limitations

This study had notable strengths, including one of the largest samples collected to date to investigate the relationship between camouflaging and mental health; this also meant that we were able to include binary gender as a moderating variable. The analyses further controlled for autistic traits and age. However, due to the small number of participants who endorsed a non-binary gender identity we were unable to conduct analyses in this subsample. Autistic adults generally report higher levels of transgender, non-binary, and gender-fluid identities than the general population [56, 61]. It is important to explore the impact of camouflaging on mental health in this group, who are already at a greater risk of mental health problems compared to cisgender individuals [62, 63]. Additionally, our sample did not include individuals who are representative of the entire autistic population; only those who were able to complete online surveys in English were able to take part. Our sample was older on average than many previous studies, had relatively high levels of education and employment, and participants were diagnosed with autism later in life. This is a strength as most research into autism and mental health focuses on those who were diagnosed early in life and may have received more support for their autism and/or mental health problems, but also means our findings may not be generalisable to autistic children and adolescents, or those who were diagnosed in early childhood.

Another limitation is that, due to the anonymous nature of online data collection, we were not able to independently verify participants’ self-reported diagnosis of either autism or mental health conditions. Due to changes over time and across countries in autism diagnostic criteria and categories, the precise diagnostic label (e.g. ‘Asperger’s syndrome’ vs. ‘childhood autism’) reported by participants might represent variation in autistic characteristics which was not captured in the present study. These findings should be replicated in clinical settings, where more information about the exact nature of participants’ autistic and mental health diagnoses and symptoms can be recorded. In particular, as the measures used to assess mental health conditions in the present study only ask about symptoms in the previous two weeks, an evaluation of participants’ longer-term psychiatric history might reveal even stronger associations between camouflaging and mental health problems.

We also did not have information on participants’ socioeconomic status, cognitive ability, or additional psychological characteristics such as social motivation or attitudes to camouflaging. These variables are all likely to influence mental health and may mediate or moderate the association between camouflaging and mental health. They should therefore be included in future research to better understand the unique contribution of camouflaging.

It is also possible that additional common causal factors (confounders) such as isolation or victimisation contribute to the simultaneous development of both mental health difficulties and camouflaging in autistic people, the latter as an attempt to form more connections and be accepted by others. These are important research and clinical questions, which can only be addressed through the follow-up of autistic individuals over time, or via experimental studies, such as randomised controlled trials of interventions designed to reduce camouflaging.

Many results reported in this manuscript were significant at a very conservative level (*p* < 0.001); however, others were significant at a more modest level (*p* < 0.05). We advise caution in interpreting these results with the same degree of confidence, such as the impact of camouflaging on depression in the total sample (Table 2, Model 2).

## Conclusions

This study found that greater self-reported camouflaging is associated with poor mental health outcomes in autistic adults. No moderating effect of gender was found, suggesting that camouflaging has similarly poor mental health consequences for autistic men and women. The association between camouflaging and poor mental health appears to be linear, with the likelihood of poor mental health increasing across the entire range of camouflaging scores. However, there may be different thresholds at which camouflaging predicts scoring above clinical cut-offs for specific mental health conditions. These thresholds may be useful for identifying those at the greatest risk of mental health problems, although we caution their use as diagnostic or deterministic cut-offs. It is imperative that causal associations between camouflaging and mental health in autism are investigated directly.

## Supplementary Information

**Additional file 1:** Bivariate correlations between all variables and hierarchical regression analyses in sample split by gender identity.

## Data Availability

The anonymised dataset is available on reasonable request from Laura Hull (laura.hull.14@ucl.ac.uk).

## Abbreviations

ADHD: Attention deficit hyperactivity disorder
BAPQ: Broad Autism Phenotype Questionnaire
CAT-Q: Camouflaging Autistic Traits Questionnaire
GAD-7: Generalised Anxiety Disorder (7 items)
LSAS: [Liebowitz] Social Anxiety Scale
ODD: Oppositional defiant disorder
PHQ-9: Patient Health Questionnaire (9 items)
