# Supplementary material for: Is social camouflaging associated with anxiety and depression in autistic adults?
Source: Mol Autism. 2021 Feb 16;12:13. doi: 10.1186/s13229-021-00421-1 (PMC7885456; doi:10.1186/s13229-021-00421-1)
Supplement: Supplementary file 1 — Additional file 1: Bivariate correlations between all variables and hierarchical regression analyses in sample split by gender identity. [file 13229_2021_421_MOESM1_ESM.docx]

Supplementary Table 1. Correlations between all variables for total sample (N = 305)

|  | Age | CAT-Q | BAPQ | LSAS | PHQ |
| --- | --- | --- | --- | --- | --- |
| CAT-Q | -0.17 (p = .003) |  |  |  |  |
| BAPQ | 0.04 (p = .441) | 0.22 (p < .001) |  |  |  |
| LSAS | -0.11 (p = .062) | 0.35 (p < .001) | 0.60 (p < .001) |  |  |
| PHQ | -0.16 (p = .005) | 0.22 (p < .001) | 0.35 (p < .001) | 0.41 (p < .001) |  |
| GAD | -0.13 (p = .025) | 0.32 (p < .001) | 0.39 (p < .001) | 0.43 (p < .001) | 0.73 (p < .001) |

CAT-Q = Camouflaging Autistic Traits Questionnaire; BAPQ = Broad Autism Phenotype Questionnaire; LSAS = Leibowitz Social Anxiety Scale; PHQ = Patient Health Questionnaire; GAD = Generalised Anxiety Disorder Assessment.

Supplementary Table 2. Correlations between all variables for Female (N = 181; below diagonal) and Male (N = 104; above diagonal) subsamples

|  | Age | CAT-Q | BAPQ | LSAS | PHQ | GAD |
| --- | --- | --- | --- | --- | --- | --- |
| Age |  | -0.09  (p = .367) | 0.12  (p = .233) | -0.08  (p = .415) | -0.15  (p = .133) | -0.12  (p = .217) |
| CAT-Q | -0.17  (p = .025) |  | 0.28  (p = .004) | 0.35  (p < .001) | 0.31  (p = .001) | 0.45  (p < .001) |
| BAPQ | 0.05  (p = .528) | 0.15  (p = .043) |  | 0.72  (p < .001) | 0.34  (p < .001) | 0.43  (p < .001) |
| LSAS | -0.06  (p = .401) | 0.29  (p < .001) | 0.51  (p < .001) |  | 0.36  (p < .001) | 0.45  (p < .001) |
| PHQ | -0.12  (p = .107) | 0.14  (p = .069) | 0.37  (p < .001) | 0.48  (p < .001) |  | 0.72  (p < .001) |
| GAD | -0.09  (p = .204) | 0.20  (p = .007) | 0.40  (p < .001) | 0.46  (p < .001) | 0.74  (p < .001) |  |

CAT-Q = Camouflaging Autistic Traits Questionnaire; BAPQ = Broad Autism Phenotype Questionnaire; LSAS = Leibowitz Social Anxiety Scale; PHQ = Patient Health Questionnaire; GAD = Generalised Anxiety Disorder Assessment.

Supplementary Table 3. Correlations between all variables for Non-Binary subsample (N = 18)

|  | Age | CAT-Q | BAPQ | LSAS | PHQ |
| --- | --- | --- | --- | --- | --- |
| CAT-Q | -0.10  (p = .684) |  |  |  |  |
| BAPQ | -0.05  (p = .835) | 0.18  (p = .482) |  |  |  |
| LSAS | -0.01  (p = .977 | 0.39  (p = .110) | 0.05  (p = .846) |  |  |
| PHQ | -0.33  (p = .189) | 0.42  (p = .085) | 0.21  (p = .399) | 0.15  (p = .551) |  |
| GAD | -0.23  (p = .356) | 0.39  (p = 0.109) | -0.06  (p = .812) | -0.07  (p = .776) | 0.65  (p = .004) |

CAT-Q = Camouflaging Autistic Traits Questionnaire; BAPQ = Broad Autism Phenotype Questionnaire; LSAS = Leibowitz Social Anxiety Scale; PHQ = Patient Health Questionnaire; GAD = Generalised Anxiety Disorder Assessment.

Supplementary Table 4. Results of multiple regression analyses in sample split by gender identity (Cisgender female N = 178, Cisgender male N = 100, transgender/non-binary N = 22).

| Model 1a (Generalised Anxiety) | | | | | | | | | |
| --- | --- | --- | --- | --- | --- | --- | --- | --- | --- |
|  | Variable | B | β | p | DF | F | p | R^2^Adj | Delta R2 |
| Step 1 |  |  |  |  | 4, 297 | 21.45 | <.001 | 0.21 |  |
|  | Age | -0.05 | -0.10 | .058 |  |  |  |  |  |
|  | Gender | -0.11 | -0.01 | .845 |  |  |  |  |  |
|  | BAPQ | 3.28 | 0.35 | <.001 |  |  |  |  |  |
|  | CAT-Q | 0.08 | 0.22 | <.001 |  |  |  |  |  |
| Step 2 |  |  |  |  | 5, 296 | 17.23 | <.001 | 0.21 | .00 |
|  | Age | -0.05 | -0.11 | .054 |  |  |  |  |  |
|  | Gender | 2.24 | 0.21 | .505 |  |  |  |  |  |
|  | BAPQ | 3.27 | 0.35 | <.001 |  |  |  |  |  |
|  | CAT-Q | 0;11 | 0.33 | .036 |  |  |  |  |  |
|  | CAT-Q * Gender | -0.02 | -0.05 | .478 |  |  |  |  |  |
| Model 2a (Depression) | | | | | | | | | |
|  | Variable | B | β | p | DF | F | p | R^2^Adj | Delta R2 |
| Step 1 |  |  |  |  | 4, 297 | 15.16 | <.001 | 0.16 |  |
|  | Age | -0.07 | -0.15 | .010 |  |  |  |  |  |
|  | Gender | 0.18 | 0.01 | .791 |  |  |  |  |  |
|  | BAPQ | 3.40 | 0.33 | <.001 |  |  |  |  |  |
|  | CAT-Q | 0.05 | 0.12 | .028 |  |  |  |  |  |
| Step 2 |  |  |  |  | 5, 296 | 12.10 | <.001 | 0.15 | .01 |
|  | Age | -0.07 | -0.15 | .001 |  |  |  |  |  |
|  | Gender | 1.14 | 0.09 | .767 |  |  |  |  |  |
|  | BAPQ | 3.40 | 0.33 | <.001 |  |  |  |  |  |
|  | CAT-Q | 0.06 | 0.16 | .317 |  |  |  |  |  |
|  | CAT-Q * Gender | -0.01 | -0.02 | .799 |  |  |  |  |  |
| Model 3a (Social Anxiety) | | | | | | | | | |
|  | Variable | B | β | p | DF | F | p | R^2^Adj | Delta R2 |
| Step 1 |  |  |  |  | 4, 297 | 53.00 | <.001 | 0.41 |  |
|  | Age | -0.18 | -0.08 | .075 |  |  |  |  |  |
|  | Gender | 2.17 | 0.04 | .365 |  |  |  |  |  |
|  | BAPQ | 24.53 | 0.55 | <.001 |  |  |  |  |  |
|  | CAT-Q | 0.34 | 0.21 | <.001 |  |  |  |  |  |
| Step 2 |  |  |  |  | 5, 296 | 42.26 | <.001 | 0.41 | 0.00 |
|  | Age | -0.18 | -0.08 | .076 |  |  |  |  |  |
|  | Gender | 2.40 | 0.05 | .864 |  |  |  |  |  |
|  | BAPQ | 24.53 | 0.55 | <.001 |  |  |  |  |  |
|  | CAT-Q | 0.35 | 0.21 | .123 |  |  |  |  |  |
|  | CAT-Q * Gender | -0.01 | -0.01 | .987 |  |  |  |  |  |

BAPQ = Broad Autism Phenotype Questionnaire; CAT-Q = Camouflaging Autistic Traits Questionnaire; β = standardised beta.
